# Uncontrolled asthma: a retrospective cohort study in Japanese patients newly prescribed with medium-/high-dose ICS/LABA

**DOI:** 10.1038/s41533-021-00222-2

**Published:** 2021-03-02

**Authors:** Hiromasa Inoue, Ki Lee Milligan, Aine McConnon, Hajime Yoshisue, Emil Loefroth, Martin McSharry, Akihito Yokoyama, Masakazu Ichinose

**Affiliations:** 1 Department of Pulmonary Medicine, Kagoshima University, Kagoshima, Japan; 2 Novartis Pharma AG, Basel, Switzerland; 3 Novartis Ireland Limited, Dublin, Ireland; 4 Novartis Pharma K.K. Tokyo, Tokyo, Japan; 5 Novartis Sverige AB, Kista, Sweden; 6 OptumRx, Dublin, Ireland; 7 Department of Respiratory Medicine and Allergology, Kochi Medical School, Kochi University, Kochi, Japan; 8 Osaki Citizen Hospital, Osaki, Japan

**Keywords:** Epidemiology, Asthma

## Abstract

Many asthma patients remain uncontrolled despite guideline-based therapies. We examined real-life asthma control in Japanese patients prescribed with inhaled corticosteroid/long-acting β_2_-agonist (ICS/LABA). Patients (≥12 years) with ≥2 asthma diagnoses, newly initiated on medium-/high-dose ICS/LABA (Japanese asthma guidelines), from 01 April 2009 to 31 March 2015 were included, using Japan Medical Data Center Claims Database. Primary objective: proportion of patients with uncontrolled asthma in the year following ICS/LABA initiation. Secondary objectives: predictors of uncontrolled asthma and healthcare resource utilization. In medium-dose (*N* = 24,937) and high-dose (*N* = 8661) ICS/LABA cohorts, 23% and 21% patients, respectively, were uncontrolled. Treatment step up and exacerbation were most common indicators of uncontrolled asthma. Predictors of uncontrolled asthma, analyzed by multivariable Cox model, included systemic corticosteroid use, exacerbation history, comorbidities, and being female. In both cohorts, healthcare resource utilization was higher in patients with uncontrolled asthma. Over 20% patients with persistent asthma who initiated medium- or high-dose ICS/LABA were uncontrolled, highlighting unmet need for novel therapies in these patients.

## Introduction

Asthma is a major chronic respiratory disease affecting >350 million people worldwide^1^. In Japan, approximately 3 million people suffer from asthma, making it the leading chronic respiratory disease nationally^2^.

Recommendations for asthma care are established by well-known clinical entities such as the Global Initiative for Asthma (GINA)^3^ report and regional Japanese asthma guidelines (JGL)^4^. Inhaled corticosteroid (ICS) is the cornerstone in asthma management and the first choice for patients requiring regular maintenance treatment^3^. For patients who experience persistent symptoms and exacerbations, guidelines recommend use of ICS with a long-acting β_2_-agonist (ICS/LABA) or a higher dose of ICS. However, a considerable number of asthma patients receiving guideline-based therapies continue to have poor disease control, diminished quality of life, reduced work productivity, and emergency or hospital-based medical care^5,6^.

This subpopulation of patients with uncontrolled asthma accounts for considerable healthcare expenditures^7^; however, there is limited evidence on the prevalence, characteristics, and burden of poor asthma control in patients with ICS/LABA prescription^6,8^. Particularly in Asia, there is a lack of data on the level of disease control following prescription with ICS/LABA, and frequency of treatment escalation when asthma remains poorly controlled, and healthcare resource utilization.

In order to understand the clinically meaningful response to ICS/LABA therapy in the Japanese population, we conducted a retrospective cohort study using the Japan Medical Data Centre (JMDC) Claims Database to identify the proportion of patients with uncontrolled asthma in the 1-year period following newly initiated treatment with medium- or high-dose ICS/LABA. Additionally, we investigated the demographics and clinical characteristics associated with uncontrolled asthma in patients treated with ICS/LABA, treatment pathways following the first sign of uncontrolled asthma, and healthcare resource utilization burden in these patients.

## Results

### Study population

**Fig. 1:**
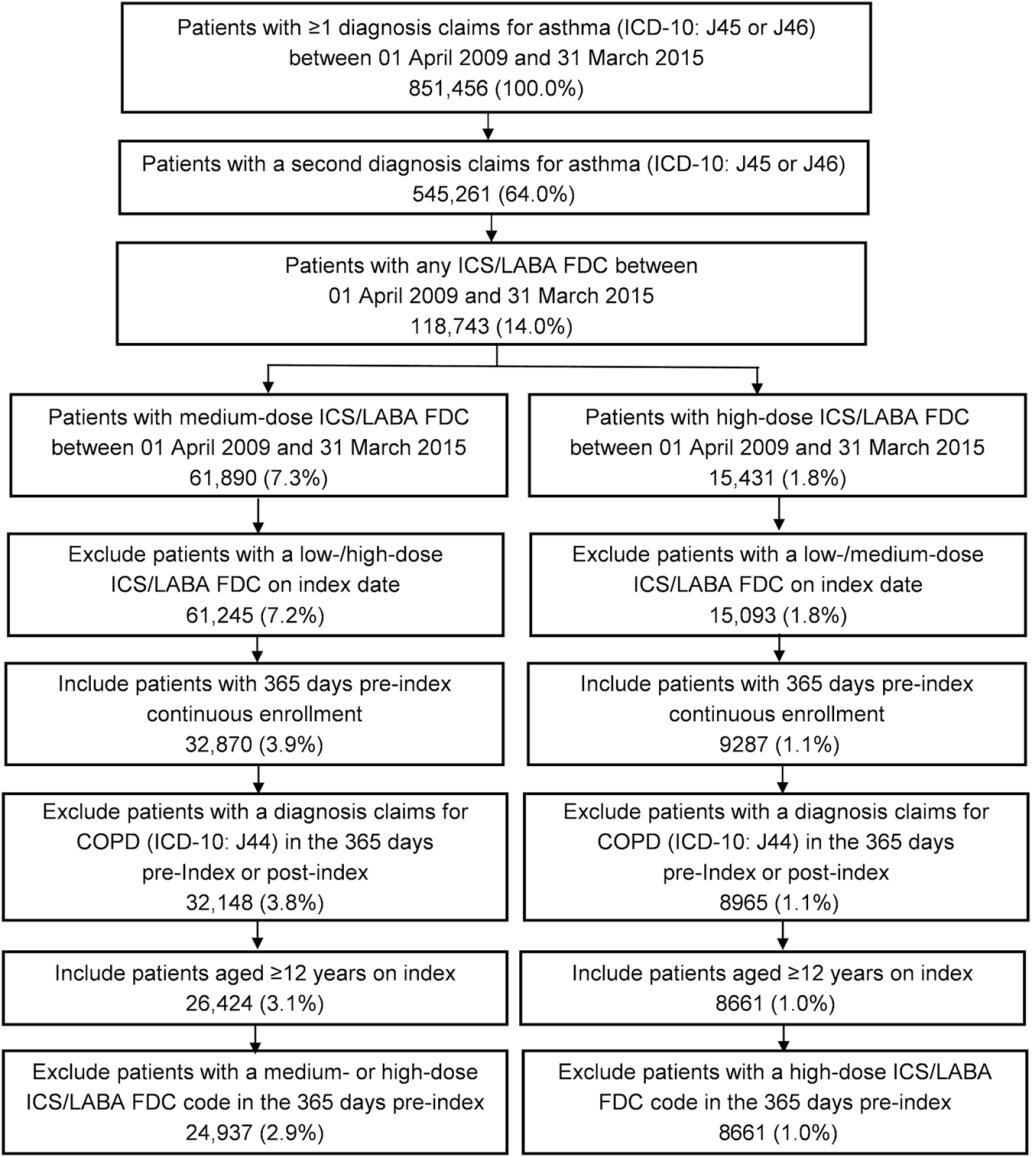
Patient disposition according to JGL^4^. COPD chronic obstructive pulmonary disease, FDC fixed-dose combination, ICS inhaled corticosteroid, JGL Japanese asthma guidelines, LABA long-acting β_2_-agonist.

A total of 24,937 patients met the inclusion criteria for the medium-dose ICS/LABA cohort and 8661 patients for the high-dose cohort based on the definition of JGL^4^. Patient disposition according to JGL-defined and GINA-defined^9^ medium- and high-dose ICS/LABA cohorts is shown in Fig. 1 and Supplementary Fig. 1, respectively.

The mean ± standard deviation (SD) age at index date was 38.9 ± 13.31 years in the medium-dose ICS/LABA cohort and 40.8 ± 11.94 years in the high-dose ICS/LABA cohort. Gender distribution was comparable between the cohorts as was body mass index (BMI). A total of 2260 patients (9.1%) in the medium-dose cohort and 840 patients (9.7%) in the high-dose cohort were habitual smokers. In the 1-year prior to the index date, 10.8% of patients in the medium-dose cohort and 15.7% of patients in the high-dose cohort experienced ≥1 exacerbation. Rhinitis/rhinosinusitis was the most common comorbidity reported in both cohorts (medium-dose cohort, 76.2%; high-dose cohort, 78.5%), with allergic rhinitis/rhinosinusitis being the most common subtype (Supplementary Table 1). Baseline demographics and clinical characteristics of patients in the GINA-defined^9^ medium- and high-dose ICS/LABA cohorts are detailed in Supplementary Table 2.

### Asthma control in the follow-up period

In the 1 year following the index date, 22.9% of patients (*n* = 5707) in the medium-dose ICS/LABA cohort and 20.7% patients (*n* = 1793) in the high-dose ICS/LABA cohort (both cohorts were defined based on JGL^4^) were found to have uncontrolled asthma (Table 1). The median number of days to first episode of uncontrolled asthma was 34 days for the medium-dose cohort and 21 days in the high-dose cohort. In the medium-dose cohort, the median number of days for first episode of asthma exacerbation was 11 days, while it was 8 days for the high-dose cohort. The reasons for uncontrolled asthma are listed in Table 1.

**Table 1:** Asthma control in medium- and high-dose ICS/LABA cohort and reasons for uncontrolled asthma control.

	Medium-dose ICS/LABA cohort (*N* = 24,937)	High-dose ICS/LABA cohort (*N* = 8661)
Patients with uncontrolled asthma	5707 (22.9%)	1793 (20.7%)
Reasons for uncontrolled asthma		
Episode of exacerbation		
Moderate exacerbation	1455 (5.8%)	676 (7.8%)
Severe exacerbation	197 (0.8%)	92 (1.1%)
Treatment step up		
ICS increased dose	442 (1.8%)	0
Addition of LAMA	10 (0.0%^a^)	11 (0.1%)
Addition of LTRA	1920 (7.7%)	495 (5.7%)
Addition of theophylline	594 (2.4%)	212 (2.5%)
Maintenance systemic corticosteroids	75 (0.3%)	31 (0.4%)
Addition of more than one asthma controller, including LTRA	198 (0.8%)	55 (0.6%)
Addition of more than one asthma controller, excluding LTRA	23 (0.1%)	0
High-dose SABA	568 (2.3%)	186 (2.2%)
Use of adrenaline	633 (2.5%)	169 (2.0%)

Data are presented as *n* (%).

*ICS* inhaled corticosteroid, *LABA* long-acting β_2_-agonist, *LAMA* long-acting muscarinic antagonist, *LTRA* leukotriene receptor antagonist, *SABA* short-acting β_2_-agonist.

^a^0.04%.

Differences in baseline characteristics between patients with controlled asthma and uncontrolled asthma are presented in Table 2 for both cohorts. Patients with uncontrolled asthma, independent of ICS/LABA dosing, were more likely to be older, with no significant differences observed in BMI. In both cohorts, patients with uncontrolled asthma were significantly more likely to have had one or more asthma exacerbation in the 1-year pre-index than patients with controlled asthma. More patients with uncontrolled asthma were prescribed asthma drugs such as leukotriene receptor antagonist (LTRA), short-acting β_2_-agonist (SABA), theophylline, and a maintenance systemic corticosteroid in the 1-year pre-index compared with controlled patients in both the cohorts. Patients with uncontrolled asthma also tended to have more comorbidities than controlled patients (Table 2).

**Table 2:** Demographic and clinical characteristics of patients achieving asthma control versus patients with uncontrolled asthma in the post-index period.

Characteristic	Medium-dose ICS/LABA cohort (*N* = 24,937)			High-dose ICS/LABA cohort (*N* = 8661)		
	Controlled asthma (*n* = 19,230)	Uncontrolled asthma (*n* = 5707)	*P* value	Controlled asthma (*n* = 6868)	Uncontrolled asthma (*n* = 1793)	*P* value
Sex						
Male	9018 (46.9%)	2480 (43.5%)	<0.0001^a^	3389 (49.3%)	793 (44.2%)	0.0001^a^
Female	10,212 (53.1%)	3227 (56.5%)		3479 (50.7%)	1000 (55.8%)	
Age at index date, years						
Mean ± SD	38.8 ± 13.26	39.4 ± 13.48	0.0038^b^	40.7 ± 11.94	41.4 ± 11.91	0.0304^b^
Range	12–75	12–75		12–75	12–74	
Median (IQR)	39.0 (30.0–48.0)	39.0 (31.0–48.0)		40.0 (33.0–48.0)	41.0 (33.0–49.0)	
BMI, kg/m^2^						
Mean ± SD	23.3 ± 4.00	23.2 ± 4.08	0.3220^b^	23.5 ± 4.07	23.6 ± 4.43	0.7950^b^
Range	13.5–54.9	14.6–46.6		15.3–52.0	14.0–42.4	
Median (IQR)	22.7 (20.5–25.3)	22.6 (20.4–25.3)		22.9 (20.7–25.6)	22.7 (20.4–25.9)	
Index year						
2010	1726 (9.0%)	615 (10.8%)	<0.0001^a^	373 (5.4%)	158 (8.8%)	<0.0001^a^
2011	3128 (16.3%)	1097 (19.2%)		702 (10.2%)	316 (17.6%)	
2012	4148 (21.6%)	1206 (21.1%)		1022 (14.9%)	284 (15.8%)	
2013	3922 (20.4%)	1142 (20.0%)		1246 (18.1%)	316 (17.6%)	
2014	5233 (27.2%)	1499 (26.3%)		2668 (38.9%)	640 (35.7%)	
2015	1073 (5.6%)	148 (2.6%)		857 (12.5%)	79 (4.4%)	
Asthma exacerbations in 1-year pre-index						
Mean ± SD	0.1 ± 0.55	0.3 ± 1.17	<0.0001^b^	0.2 ± 0.69	0.6 ± 1.70	<0.0001^b^
Range	0–12	0–23		0–16	0–25	
Median (IQR)	0 (0–0)	0 (0–0)		0 (0–0)	0 (0–0)	
Patients with asthma exacerbations in 1-year pre-index						
0	17,460 (90.8%)	4780 (83.8%)	<0.0001^b^	5967 (86.9%)	1335 (74.5%)	<0.0001^b^
≥1	1770 (9.2%)	927 (16.2%)		901 (13.1%)	458 (25.5%)	
Asthma drugs in 1-year pre-index						
LAMA	35 (0.2%)	16 (0.3%)	0.1487^a^	21 (0.3%)	10 (0.6%)	0.1116^a^
LTRA	5949 (30.9%)	1929 (33.8%)	<0.0001^a^	2649 (38.6%)	763 (42.6%)	0.0021^a^
SABA	3145 (16.4%)	1121 (19.6%)	<0.0001^a^	1553 (22.6%)	547 (30.5%)	<0.0001^a^
Theophylline	2927 (15.2%)	992 (17.4%)	<0.0001^a^	1399 (20.4%)	465 (25.9%)	<0.0001^a^
Maintenance systemic corticosteroids	34 (0.2%)	83 (1.5%)	<0.0001^a^	22 (0.3%)	32 (1.8%)	<0.0001^a^
Concomitant medications in 1-year pre-index						
NSAIDs	3831 (19.9%)	1373 (24.1%)	<0.0001^a^	1582 (23.0%)	477 (26.6%)	0.0016^a^
Beta-blockers	300 (1.6%)	103 (1.8%)	0.1979^a^	102 (1.5%)	37 (2.1%)	0.0826^a^
Acetaminophen	6056 (31.5%)	1952 (34.2%)	0.0001^a^	2022 (29.4%)	586 (32.7%)	0.0077^a^
Comorbidities						
Atrial fibrillation or other cardiac arrhythmias	1218 (6.3%)	466 (8.2%)	<0.0001^a^	540 (7.9%)	150 (8.4%)	0.4834^a^
Anaphylaxis	59 (0.3%)	39 (0.7%)	<0.0001^a^	31 (0.5%)	14 (0.8%)	0.0840^a^
Eczema	8238 (42.8%)	2636 (46.2%)	<0.0001^a^	3095 (45.1%)	871 (48.6%)	0.0078^a^
GERD	2685 (14.0%)	1001 (17.5%)	<0.0001^a^	1323 (19.3%)	394 (22.0%)	0.0103^a^
Heart failure	833 (4.3%)	340 (6.0%)	<0.0001^a^	404 (5.9%)	113 (6.3%)	0.5039^a^
Rhinitis/rhinosinusitis	14,428 (75.0%)	4563 (80.0%)	<0.0001^a^	5355 (78.0%)	1443 (80.5%)	0.0213^a^
Rhinitis/rhinosinusitis—allergic	13,053 (67.9%)	4213 (73.8%)	<0.0001^a^	4895 (71.3%)	1339 (74.7%)	0.0042^a^
Charlson Comorbidity Index						
Mean ± SD	2.2 ± 1.79	2.4 ± 2.02	<0.0001^b^	2.4 ± 1.97	2.6 ± 2.23	0.0035^b^
Range	0–17	0–16		0–16	0–16	
Median (IQR)	1.0 (1.0–3.0)	2.0 (1.0–3.0)		2.0 (1.0–3.0)	2.0 (1.0–3.0)	

Data presented as *n* (%), unless otherwise specified.

*BMI* body mass index, *FDC* fixed-dose combination, *GERD* gastroesophageal reflux disease, *ICS* inhaled corticosteroid, *IQR* interquartile range, *LABA* long-acting β_2_-agonist, *LAMA* long-acting muscarinic antagonist, *LTRA* leukotriene receptor antagonist, *NSAID* nonsteroidal anti-inflammatory drug, *SABA* short-acting β_2_-agonist.

^a^Chi-square test.

^b^Wilcoxon rank-sum test.

Predictors of uncontrolled asthma in both ICS/LABA cohorts are described in Table 3. A total of 14 variables in the medium-dose ICS/LABA cohort and 8 variables in the high-dose ICS/LABA cohort were found to be significantly associated with a risk of uncontrolled asthma, of which maintenance corticosteroid use carried the highest risk, followed by prior history of asthma exacerbation, irrespective of ICS dose. Also, index date was shown to significantly impact patients’ risk of uncontrolled asthma, with patients with an index year of 2012 (hazard ratio (HR) = 0.79, 95% confidence interval (CI): 0.72, 0.87), 2013 (HR = 0.75, 95% CI: 0.68, 0.83), 2014 (HR = 0.82, 95% CI: 0.74, 0.90), or 2015 (HR = 0.75, 95% CI: 0.63, 0.90) significantly less likely to be uncontrolled when compared with patients with an index year of 2010. Among patients in the medium- and high-dose ICS/LABA cohorts, female patients had higher risk of poor asthma control compared with males.

**Table 3:** Predictors of uncontrolled asthma in medium- and high-dose ICS/LABA cohorts.

Predictors of uncontrolled asthma	Medium-dose ICS/LABA cohort (*N* = 24,937)		High-dose ICS/LABA cohort (*N* = 8661)	
	HR (95% CI)	*P* value	HR (95% CI)	*P* value
Sex				
Female versus male	1.131 (1.073, 1.193)	<0.0001	1.232 (1.122, 1.353)	<0.0001
Index year				
2011 versus 2010	0.958 (0.867, 1.057)	0.3918	1.004 (0.830, 1.216)	0.9640
2012 versus 2010	0.790 (0.716, 0.872)	<0.0001	0.697 (0.573, 0.848)	0.0003
2013 versus 2010	0.754 (0.682, 0.834)	<0.0001	0.616 (0.508, 0.747)	<0.0001
2014 versus 2010	0.819 (0.744, 0.902)	<0.0001	0.638 (0.534, 0.762)	<0.0001
2015 versus 2010	0.750 (0.625, 0.901)	0.0020	0.449 (0.342, 0.591)	<0.0001
Asthma drugs (yes versus no)				
SABA	1.076 (1.005, 1.153)	0.0368	1.176 (1.055, 1.311)	0.0034
Maintenance systemic corticosteroids	4.705 (3.769, 5.873)	<0.0001	3.429 (2.411, 4.877)	<0.0001
Number of asthma exacerbations 1-year pre-index (≥1 versus 0)	1.666 (1.546, 1.796)	<0.0001	1.790 (1.592, 2.013)	<0.0001
Concomitant medications (yes versus no)				
NSAIDs	1.101 (1.034, 1.173)	0.0027	NA	
Comorbidities (yes versus no)				
Atrial fibrillation or other cardiac arrhythmias	1.160 (1.051, 1.282)	0.0033	NA	
Anaphylaxis	1.617 (1.179, 2.218)	0.0029	NA	
Eczema	1.072 (1.016, 1.132)	0.0115	NA	
GERD	1.179 (1.098, 1.266)	<0.0001	NA	
Heart failure	1.157 (1.030, 1.301)	0.0142	NA	
Rhinitis/rhinosinusitis	0.996 (0.881, 1.126)	0.9521	NA	
Charlson Comorbidity Index groups				
≥2 versus ≤1	1.049 (0.992, 1.109)	0.0951	NA	

Harrell’s *C*-statistic for the model: 0.90.

*GERD* gastroesophageal reflux disease, *ICS* inhaled corticosteroid, *LABA* long-acting β_2_-agonist, *NA* not applicable, *NSAID* non-steroidal anti-inflammatory drug, *SABA* short-acting β_2_-agonist.

### Treatment pathways following the first episode of uncontrolled asthma

#### Medium-dose ICS/LABA cohort

**Fig. 2:**
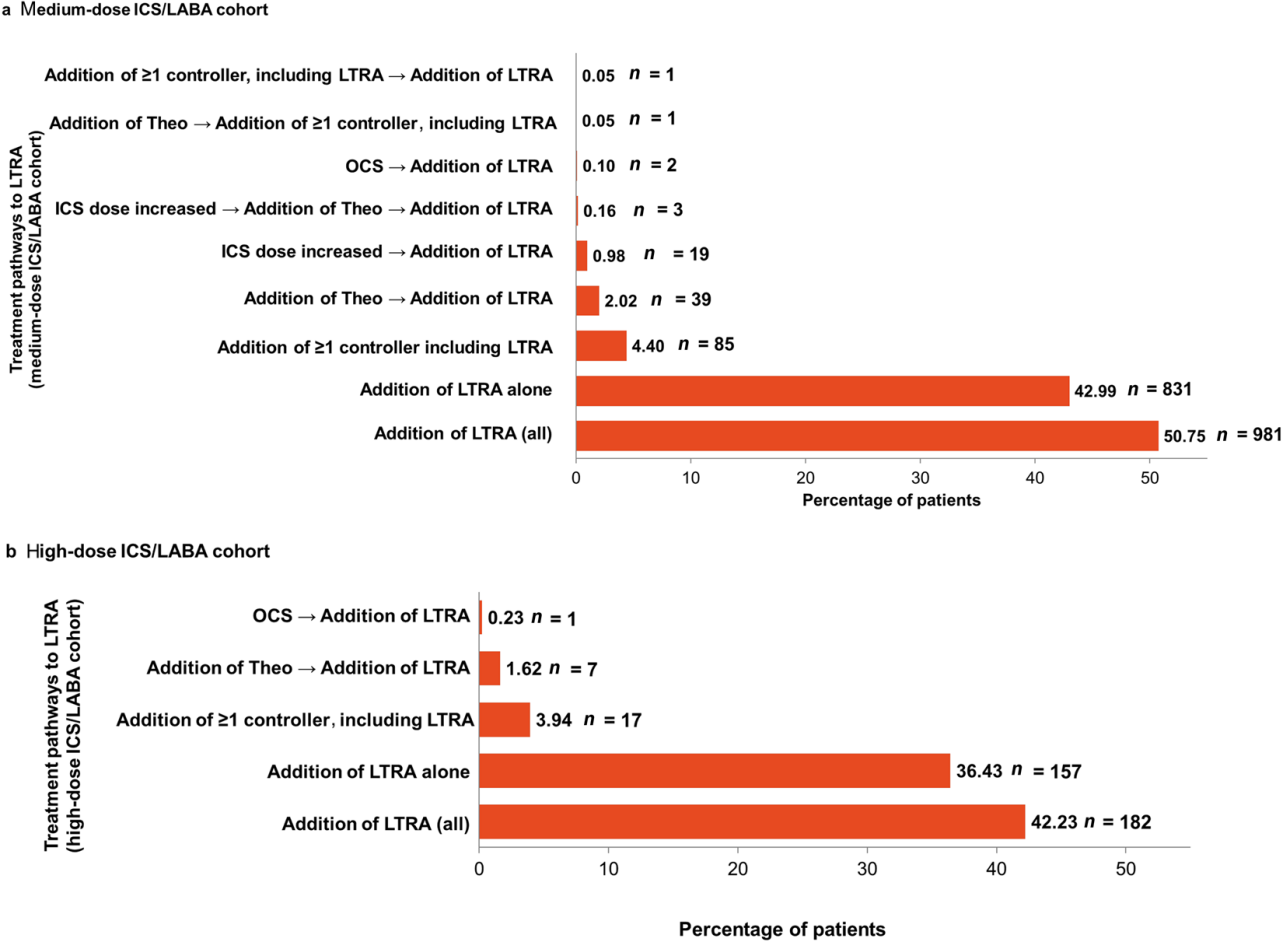
Treatment pathways to LTRA after the first episode of uncontrolled asthma in medium- and high-dose ICS/LABA cohorts. **a** Medium-dose ICS/LABA cohort; total number of patients with uncontrolled asthma and at least 365 days of follow-up, 1933; **b** high-dose ICS/LABA cohort; total number of patients with uncontrolled asthma and at least 365 days of follow-up, 431. *n* patients with treatment pathways to LTRA, ICS inhaled corticosteroid, LABA long-acting β_2_-agonist, LTRA leukotriene receptor antagonist, OCS oral corticosteroid, Theo theophylline.

A total of 624 patients (32.0%) with uncontrolled asthma did not receive treatment step up following the first event indicating loss of asthma control. Of the patients who stepped up, the prescription trend for first step up was: addition of LTRA, 43.0%; addition of theophylline, 12.1%; increased ICS dose, 6.6%; addition of more than one asthma controller including LTRA, 4.4%; maintenance corticosteroids, 1.1%; addition of more than one asthma controller excluding LTRA, 0.4%; and addition of long-acting muscarinic antagonist (LAMA), 0.2%. Second and third lines of step up medications were also evaluated. The large majority of patients received no further treatment step up (between 76% and 100%) at the second line; however, of those who did, addition of LTRA was generally the most common. Treatment pathways to LTRA after the first episode of uncontrolled asthma in the medium-dose ICS/LABA cohort are described in Fig. 2a.

#### High-dose ICS/LABA cohort

A total of 176 patients (40.8%) with uncontrolled asthma and an additional 1 year of follow-up had no treatment step up following the first episode of uncontrolled asthma. Of the patients who stepped up, the prescription trend for first step up was: addition of LTRA, 36.4%; addition of theophylline, 15.6%; addition of more than one asthma controller including LTRA, 3.9%; maintenance corticosteroids, 2.8%; and addition of LAMA, 0.5%. Second and third lines of step-up medications were also examined, again with the large majority of patients having no further step up. Figure 2b describes treatment pathways to LTRA after the first episode of uncontrolled asthma in the high-dose ICS/LABA cohort.

#### Healthcare resource utilization

Overall, the rates of asthma- and non-asthma specific healthcare resource utilization events tended to be lower in the medium-dose ICS/LABA cohort, compared with the high-dose ICS/LABA cohort. In the medium-dose cohort, patients with uncontrolled asthma had significantly higher incidence rates for all asthma- and non-asthma-specific healthcare resource utilization items compared with patients with controlled asthma (Supplementary Table 3). A similar pattern of incidence rates was also observed in the high-dose cohort (Supplementary Table 3).

Healthcare resource utilization costs were examined for the medium- and high-dose ICS/LABA cohorts. The majority of healthcare resource utilization costs were higher for uncontrolled patients compared with controlled patients for both medium- and high-dose ICS/LABA cohorts, with the difference most pronounced for asthma-specific healthcare resource utilization items and the total costs including non-asthma-related costs (Supplementary Table 4).

#### Asthma control according to GINA

Applying GINA criteria^9^, a total of 23,682 and 8600 patients were classified within the medium- and high-dose ICS/LABA cohort, respectively (patient disposition is shown in Supplementary Fig. 1). GINA and JGL categories had similar proportion of patients with uncontrolled asthma: 23.0% (*n* = 5437) in the medium-dose and 20.7% (*n* = 1782) in the high-dose cohort (Supplementary Table 5). Baseline characteristics associated with uncontrolled asthma in the GINA-defined^9^ cohorts (Supplementary Table 6) were comparable with those in the JGL^4^ cohorts (Table 2).

## Discussion

The burden of uncontrolled asthma remains in spite of numerous available medication options, established clinical care guidelines, and a rapidly evolving understanding of the disease. This was a non-interventional, retrospective cohort study in patients with asthma in Japan, using healthcare claims data, to evaluate asthma control in patients categorized into medium- and high-dose ICS/LABA cohorts according to JGL^4^ recommendations. The results of this study showed that 23.0% of patients in the medium-dose and 21.0% of patients in the high-dose cohort were uncontrolled within 1 year of newly initiating treatment with ICS/LABA. Repeating the analysis with GINA step definitions^9^, to frame the data in a global perspective, the findings remain consistent. Residents in Japan are free to choose their own healthcare providers as well as their frequency of treatment regardless of the health insurance scheme they are enrolled. According to this “Free Access System,” residents are able to receive necessary medical services in case of sickness or injuries, as long as they hold proof of insurance^10^. Despite the nation’s universal healthcare infrastructure, the results from this study represent a persisting unmet need for asthma in Japan.

In a study evaluating disease control in German patients with self-reported asthma (*n* = 382), 55.8% had not well-controlled asthma, which was defined as the asthma control test (ACT) score <20^8^; this proportion was higher compared with that observed in the current study. Similarly, in a cross-sectional analysis of patients with asthma treated with ICS/LABA in the UK, approximately two-third of the population (*n*/*N* = 452/701) were not well controlled (ACT < 20)^6^. In a retrospective cohort study using the UK-Clinical Practice Research Datalink database^11^, in which uncontrolled asthma was defined similarly to this study (episode of exacerbation, treatment step up, or above threshold SABA use), 35.1% of patients in the medium-dose ICS/LABA cohort (*N* = 29,229) and 45.7% of patients in high-dose cohort (*N* = 16,575) remained uncontrolled during 1 year after the initiation of treatment with medium- or high-dose ICS/LABA. These results suggest that Japanese asthma patients newly initiated on medium- or high-dose ICS/LABA may tend to be better controlled compared with those in other countries like Germany and the UK. However, caution needs to be exercised in comparing disease control between studies as the way in which asthma control is measured tend to vary between studies.

The present study found that patients with uncontrolled asthma tended to be older, female, more likely to have had at least one pre-index exacerbation, more likely to be prescribed a range of medications including maintenance systemic corticosteroids, as well as more likely to have a range of additional comorbidities. The factors associated with uncontrolled asthma in this study were comparable to those observed in the UK study, where age, gender, exacerbation history, baseline asthma medications including maintenance oral corticosteroids, and comorbidities (except for rhinitis) were associated with uncontrolled asthma^11^. However, there are some differences between the two studies. The frequency of exacerbation in the 1-year pre-index period was lower in the current study compared with the UK study; in the medium-dose ICS/LABA cohort in the current study, the mean number of exacerbations was 0.1 and 0.3 in the controlled and uncontrolled asthma cohorts, respectively, compared with 0.3 and 0.6 in the UK study. Similarly, in the high-dose ICS/LABA cohort in the current study, it was 0.2 and 0.6 in the controlled and uncontrolled asthma cohorts, respectively compared with 0.4 and 0.8 in the UK study^11^. Although baseline SABA use was associated with uncontrolled asthma in both studies, the frequency of SABA use was very different; >80% of patients were using SABA as one of the baseline asthma medications in the UK study, compared to 30% in the current study. Though caution is needed to compare the results of these two studies, lower exacerbation frequency and lower SABA use may implicate relatively better control in Japanese patients with asthma compared with those in the UK.

In general, these findings concur with the evidence from the previous reports on patient-related risk factors for uncontrolled asthma^9,12,13^. These findings suggest that patients with uncontrolled asthma represent a more medically “at-risk” group, being older, having more comorbidities, and a higher rate of exacerbations. Therefore, uncontrolled asthma is associated with susceptibility for negative health consequences.

In terms of indicators for uncontrolled asthma in these two cohorts, a treatment step up with LTRA or presentation with moderate exacerbation were the two most frequent reasons identified in both cohorts. It has been suggested that the popularity of LTRA as an add-on treatment for asthma, over other add-on treatment options, may be due to the Japanese patient preference for oral medications over inhaled agents^14^.

For both ICS/LABA cohorts, the predictors suggesting the greatest risk of uncontrolled asthma were prescription of maintenance systemic corticosteroids and evidence of asthma exacerbation in the pre-index period. Evidence of these two factors in the pre-index period may be suggestive of poor asthma control or severe asthma. The association between asthma exacerbation and poor asthma control is well documented^12,15^, with uncontrolled asthma more likely in those patients with more frequent exacerbations^16^. Exacerbation rate is known to increase with disease severity^9,16^ and a history of exacerbations is predictive of an increased risk of subsequent exacerbations^17^. Being able to identify and subsequently target patients at risk of exacerbations is likely to lead to more effective asthma management. Index year was also shown to significantly impact patients’ risk of uncontrolled asthma, suggesting that asthma patients in earlier years (e.g., 2010) were more likely to be uncontrolled. This may reflect the introduction, in Japan, of different ICS/LABAs (e.g., budesonide/formoterol, fluticasone/formoterol, fluticasone/vilanterol) during the timeframe of 2010 to 2015 (only fluticasone/salmeterol was available before 2010), and therefore asthma patients in subsequent years may have been better controlled as a result. In the present study, female sex was found to be a significant predictor of uncontrolled asthma in both ICS/LABA cohorts. This is in line with previous studies which have shown that female sex was associated with increased risk of time-to-first exacerbations^18,19^. Factors such as greater susceptibility to allergen triggers and increased prevalence of comorbidities have been suggested to contribute to poor symptom control in female patients with asthma compared to male patients^19,20^.

Treatment pathways in patients who were found to be uncontrolled revealed that 32% of the medium-dose and 41% of the high-dose ICS/LABA cohort had no treatment step up or add-on despite their asthma being uncontrolled. The study was not designed to capture a wider range of intervention possibilities, such as non-prescription therapies or anti-infectives, and this observation likely represents an incomplete picture of actual clinical practices, including, but not limited to, difference in perception of disease control between patients and physicians, lack of proper communication between patient and physician, etc. Moreover, in patients who received treatment step up, LTRA was the most frequent prescribed add-on medication, which might be due to the prevalent use of LTRA as add-on asthma medication in Japan^21^.

Rates of healthcare resource utilization events and associated healthcare costs were higher in the high-dose ICS/LABA cohort compared with medium-dose cohort and for uncontrolled patients versus controlled patients. These findings were in line with previous findings with healthcare resource utilization being high in asthma patients with higher severity and poorer control^6,22^.

The use of secondary databases such as JMDC for research has demonstrated utility in assessing drug utilization in various disease areas^2,23,24^ while acknowledging the purpose of health insurance claims are not for clinical research and may omit relevant clinical parameters. Several limitations of the claims database include: frequently missing data for height, weight, and tobacco smoking history; lack of primary diagnosis specification for patient encounters reporting multiple diagnoses codes; under-representation of senior aged and unemployed adults; and missing data for laboratory, spirometry information, and Single Inhaler Maintenance and Reliever Therapy use as rescue therapy. Moreover, the JMDC database includes Japanese company employees and their family members and therefore representation over 65 years old is very limited (nearly 3%)^25^. In addition, patient’s prescription compliance and persistence and inhaler technique were not available for analysis as predictors of control. According to the definition of adherence based on time over which a patient continues treatment or continues to refill the prescription, from starting to stopping therapy^26^, every patient in this study was considered adherent until the first episode of treatment discontinuation (a gap of >90 days between subsequent medium- or high-dose ICS/LABA prescription) during the post-index date. Also, data pertaining to patient-reported outcomes and objective asthma control score (e.g., ACQ score, ACT score, quality of life and symptoms) and lung function parameters were not available in the JMDC database. Therefore, the level of poor asthma control may be underestimated in this study. On the other hand, it is possible to overestimate medication use, since in most pharmaco-epidemiological studies conducted using routinely collected health data such as this study, drug prescription is used as a proxy for medication use. The performance of the model was assessed using Harrell’s *C*-statistic, which does not test for proportionality. An article by Stensrud and Hernan highlighted some of the issues of testing proportionality in medical studies, such as this. The authors explain that, due to the nature of the data in medical studies, it is likely that they may contradict the proportional hazard assumption^27^. The large sample size included in the analysis could have led to statistically significant difference between the evaluated parameters even with a difference in mean values, which may not be necessarily clinically meaningful. The study results should be interpreted with clinical perspectives.

Real-world evidence on the disease control status of patients with asthma in Japan is expected to improve the understanding of a challenging yet urgently important topic among physicians and health authorities and thus support the adoption of new modalities and policies in clinical practice to achieve better clinical outcomes.

Despite universal health access in Japan, disease control appears uncontrolled in >20% patients with asthma who initiated treatment with medium- or high-dose ICS/LABA, highlighting unmet medical needs and opportunity for novel therapies in these patients.

## Methods

### Study design and data source

This was a retrospective cohort study in patients with asthma in Japan, using healthcare claims data from the JMDC Claims Database. The JMDC Claims Database is a national, anonymized claims database of Japanese company employees and their family members, regardless of the types of medical institutions and primary or secondary healthcare use. It includes claims from around 5.6 million insured Japanese patients (as of June 2018), representative of approximately 4.4% of the total Japanese population^28,29^. The data are patient level, anonymized, and longitudinal. The database includes information on age, gender, period of healthcare coverage, healthcare claims, disease diagnoses (standard disease codes), and costs associated with healthcare claims^29^. The study period was between 01 April 2008 and 31 March 2016. Patients with ≥2 asthma diagnoses who were newly initiated on ICS/LABA during the identification period between 01 April 2009 and 31 March 2015 and fulfilled the inclusion and exclusion criteria (section below) were enrolled, and divided into two cohorts—medium- or high-dose ICS/LABA treatment cohort, according to JGL (Supplementary Table 7)^4^. Minimum of two asthma diagnoses codes at different dates with at least 30 days apart is considered to increase robustness of asthma diagnosis by avoiding intermittent asthma and including persistent asthma^2^. The index date was defined as the date of the first medium- or high-dose ICS/LABA prescription in the identification period.

**Fig. 3:**
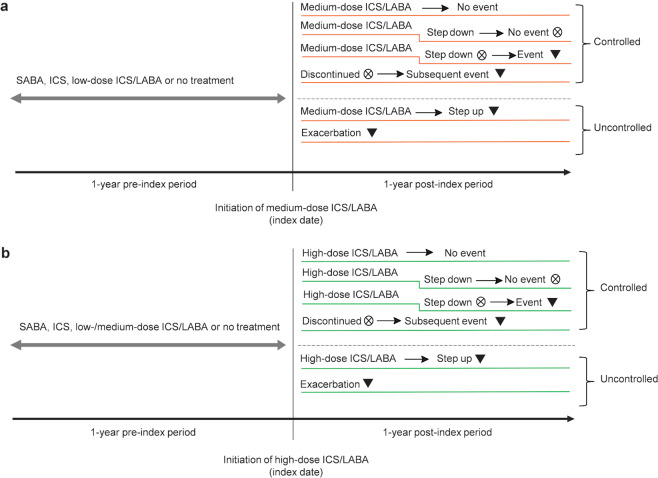
Study design for medium- and high-dose ICS/LABA cohorts. **a** Medium-dose ICS/LABA cohort; **b** high-dose ICS/LABA cohort. If no events occurred during the post-index period or if the first event happened after the patient stepped down or discontinued treatment, the patient was considered to be controlled based on asthma control achieved during the index treatment dose. Crossed circle (⊗), treatment discontinuation defined as no treatment prescription for 90 days after the runout date of last ICS/LABA prescription or step down. Inverted triangle (▾), first event of exacerbation or a treatment step up (increase in ICS dose [medium-dose ICS/LABA cohort only] or addition of another asthma controller: LAMA, LTRA, theophylline, or maintenance systemic corticosteroids). ICS inhaled corticosteroid, LABA long-acting β_2_-agonist, LAMA long-acting muscarinic antagonist, LTRA leukotriene antagonist, SABA short-acting β_2_-agonist.

Patients were followed for up to 1 year post-index date. The first event indicating uncontrolled asthma was evaluated during this period, classifying patients as either controlled or uncontrolled in the medium- and high-dose ICS/LABA cohorts. Study design for patients initiated on medium- or high-dose ICS/LABA are presented in Fig. 3.

All study data were accessed using techniques compliant with the Health Insurance Portability and Accountability Act of 1996; therefore, there was no extraction of identifiable protected health information during the course of the study, and this study did not meet requirements for informed consent from patients. This study was approved by Institutional Review Board of the Clinical Research Review Network of Japan, 1-4-9, Itachibori, Nishi-ku, Osaka, 550-0012, Japan.

### Patients

#### Inclusion criteria

Eligible patients were aged ≥12 years at the index date with the following: evidence of ≥2 claims of asthma diagnoses as defined by the International Classification of Diseases (ICD-10) of J45 or J46 occurring between April 2009 and March 2015, newly initiated on maintenance treatment with medium- or high-dose ICS/LABA between 01 April 2009 and 31 March 2015, and at least 1 year of enrollment (continuous data available in the database) prior to the index date.

#### Exclusion criteria

Patients with a diagnosis of COPD (ICD-10 J44) were excluded. Patients were excluded from the medium-dose ICS/LABA cohort if they had a prescription for medium- or high-dose ICS/LABA in the pre-index period or a prescription for low- or high-dose ICS/LABA on the index date. Patients were excluded from the high-dose ICS/LABA cohort if they had a prescription for high-dose ICS/LABA in the pre-index period or a prescription for low- or medium-dose ICS/LABA on the index date.

### Objectives and data variables

The primary objective of the study was to identify the proportion of patients with uncontrolled asthma in the 1-year period following newly initiated treatment with medium- or high-dose ICS/LABA, based on JGL. Secondary objectives were to describe and compare demographic and clinical characteristics of patients with controlled and uncontrolled asthma on medium- or high-dose ICS/LABA treatment in the 1-year period after the index date, to identify predictors of uncontrolled asthma in both study cohorts, and to describe treatment pathways following the first sign of uncontrolled asthma in both the cohorts. Other secondary endpoints included healthcare resource utilization in controlled and uncontrolled patient groups in the 1-year follow-up period after the index date and percentage of patients with uncontrolled asthma in the 1-year period after initiating treatment with a medium- or high-dose ICS/LABA fixed-dose combination, based on the GINA report (Supplementary Table 8)^9^.

Patient demographics, BMI, smoking status, use of asthma drugs, number of asthma exacerbations, concomitant medications, comorbidities, and Charlson Comorbidity Index in the pre-index period were examined.

### Definition of uncontrolled asthma

Uncontrolled asthma was defined by evidence of any of the following in the post-index period: moderate or severe exacerbation, treatment step up, above threshold SABA use or adrenaline use (Table 4). Those who discontinued (no treatment prescription for 90 days after the runout date of last ICS/LABA prescription) or stepped down from the respective (medium or high) dose ICS/LABA and then had any event were pre-defined as “controlled,” because those were considered as controlled with the respective (medium or high) dose of ICS/LABA before discontinuation or step down.

**Table 4:** Uncontrolled asthma outcomes.

Outcome	Definition
Episode of exacerbation	• Moderate exacerbation was defined as an outpatient claim for asthma plus a systemic corticosteroids burst claim of ≤30 days (within 7 days of the outpatient claim)^4,30^ • Severe exacerbation was defined as hospitalization and/or ER visit claim for asthma
Treatment step up^a^	• Increased ICS dose (for the medium-dose ICS/LABA patient cohort only) or addition of another asthma controller: LAMA, LTRA, theophylline, or maintenance systemic corticosteroids^b^
Above threshold use of SABA^c^	• An average daily dose >2 puffs/day of salbutamol equivalent (i.e., >200 µg/day for inhaled salbutamol and >20 µg/day for inhaled procaterol)^31,32^
Use of adrenaline	Identified by any claim of its use in the form of Bosmin^®^ or Epipen^®^

*ER* emergency room, *ICS* inhaled corticosteroid, *LABA* long-acting β_2_-agonist, *LAMA* long-acting muscarinic antagonist, *LTRA* leukotriene receptor antagonist, *SABA* short-acting β_2_-agonist.

^a^Indication of a treatment step up was the first claim in the post-index period for a given drug, with no evidence of that same drug in the previous 90 days.

^b^Maintenance systemic corticosteroids was defined as any systemic corticosteroids prescription with ≥30 days of supply.

^c^The first claim in the post-index period for SABA (at or above the stated thresholds), with no evidence of that same drug (at or above the stated thresholds) in the previous 90 days.

Treatment step down was identified as decreased ICS/LABA dose or step down to single controller such as ICS alone in the post-index period. Treatment pathways were described following the first episode of uncontrolled asthma as: increased ICS dose (for medium-dose cohort), addition of LAMA, addition of LTRA, addition of maintenance systemic corticosteroids, addition of more than one asthma controller in the same day, including LTRA, or addition of more than one asthma controller in the same day, excluding LTRA.

Healthcare resource utilization was evaluated in the follow-up period based on the number of outpatient visit, hospitalizations, outpatient emergency room (ER), length of stay in hospitalization, and prescriptions^2^. All healthcare resource utilization variables were assessed for both asthma specific and all cause (excluding asthma specific). Healthcare costs were calculated based on ER visit cost, inpatient cost, outpatient cost, and outpatient prescription drug cost.

### Statistical analysis

Categorical variables are reported as frequency (*n*) and proportion (%) of total patients having the given characteristics; continuous variables are summarized with the mean, standard deviation (SD), median, 25^th^ percentile, 75^th^ percentile, interquartile range, minimum, and maximum. Categorical variables were compared using chi-square tests, while continuous variables were compared using Wilcoxon rank test. No imputation methods were used in this study and complete case analysis was used in the Cox regression analysis.

The frequency and proportion of patients with uncontrolled asthma in the post-index period are reported separately for medium- and high-dose ICS/LABA cohorts. The comparison of demographics and baseline characteristics between patients who achieved asthma control and those who did not achieve asthma control was performed using univariate tests.

Predictors of uncontrolled asthma were identified based on evaluation of occurrence of poor control; adjusted HRs and 95% CIs for risk of time-to-first episode of uncontrolled asthma were estimated using a multivariable Cox model. Harrell’s *C*-statistic was calculated to assess the performance of the model. Treatment pathways were calculated on the subset of patients with an episode of uncontrolled asthma, whether or not this episode was a change of treatment. Among this subgroup, the frequency and proportion of patients is reported for each of the identified treatment pathways or treatment transitions following the first episode of uncontrolled asthma for the medium- or high-dose ICS/LABA cohort.

Healthcare resource utilization was assessed during the follow-up period by evaluating the rates of healthcare resource utilization events (outpatient visit, hospitalizations, outpatient ER, length of stay in hospitalization, and prescriptions; asthma specific and all cause [excluding asthma specific]), using univariate analysis. All healthcare resource utilization-related rates were divided by the patient-specific days of follow-up. Rate and 95% CI were calculated with SAS procedure GENMOD, using Poisson distribution, with log (follow-up) as offset, and Wald test was used to compare rates between controlled versus uncontrolled asthma. Annualized healthcare costs were reported using the following calculation: costs during follow-up period/(duration of follow-up period/365), and Wilcoxon rank test was used to compare costs between controlled versus uncontrolled asthma. All analyses were performed using SAS version 9.4.

### Reporting summary

Further information on research design is available in the Nature Research Reporting Summary linked to this article.

## Supplementary information

Supplementary Information

Reporting Summary

## Data Availability

Novartis is committed to sharing access to patient-level data and supporting documents from eligible studies with qualified external researchers. These requests are reviewed and approved by an independent review panel on the basis of scientific merit. All data provided are anonymized to respect the privacy of patients who have participated in the trial in line with applicable laws and regulations.

## References

[1] GBD Chronic Respiratory Disease Collaborators. (2017). Global, regional, and national deaths, prevalence, disability-adjusted life years, and years lived with disability for chronic obstructive pulmonary disease and asthma, 1990-2015: a systematic analysis for the Global Burden of Disease Study 2015. Lancet Respir. Med..

[2] Inoue H (2019). A retrospective cohort study evaluating healthcare resource utilization in patients with asthma in Japan. NPJ Prim. Care Respir. Med..

[3] Global Initiative for Asthma. Global strategy for asthma management and prevention. www.ginasthma.org (2018).

[4] Ichinose M (2017). Japanese guidelines for adult asthma 2017. Allergol. Int..

[5] Partridge MR, van der Molen T, Myrseth SE, Busse WW (2006). Attitudes and actions of asthma patients on regular maintenance therapy: the INSPIRE study. BMC Pulm. Med..

[6] Pavord ID (2017). The impact of poor asthma control among asthma patients treated with inhaled corticosteroids plus long-acting beta2-agonists in the United Kingdom: a cross-sectional analysis. NPJ Prim. Care Respir. Med..

[7] Lommatzsch M, Virchow JC (2014). Severe asthma: definition, diagnosis and treatment. Dtsch. Arztebl. Int..

[8] Kondla A, Glaab T, Pedersini R, Lommatzsch M (2016). Asthma control in patients treated with inhaled corticosteroids and long-acting beta agonists: a population-based analysis in Germany. Respir. Med..

[9] Global Initiative for Asthma. Global strategy for asthma management and prevention. www.ginasthma.org (2016).

[10] Japan Health Policy Now (JHPN). Japan Health Policy. http://japanhpn.org/en/jhpn/ (2020).

[11] Buhl R (2020). One-year follow up of asthmatic patients newly initiated on treatment with medium- or high-dose inhaled corticosteroid-long-acting beta2-agonist in UK primary care settings. Respir. Med..

[12] Price D (2016). Predicting frequent asthma exacerbations using blood eosinophil count and other patient data routinely available in clinical practice. J. Asthma Allergy.

[13] Wechsler ME (2014). Getting control of uncontrolled asthma. Am. J. Med..

[14] Morikawa A (2006). Asthma: what we learn from each other’s problems? The present state of asthma in Japan and Japanese Pediatric Guidelines for the Treatment and Management of Asthma (JPGTMA). Paediatr. Respir. Rev..

[15] Kupczyk M (2014). Frequent exacerbators–a distinct phenotype of severe asthma. Clin. Exp. Allergy.

[16] Suruki RY, Daugherty JB, Boudiaf N, Albers FC (2017). The frequency of asthma exacerbations and healthcare utilization in patients with asthma from the UK and USA. BMC Pulm. Med..

[17] Miller MK, Lee JH, Miller DP, Wenzel SE, Tenor Study Group. (2007). TRecent asthma exacerbations: a key predictor of future exacerbations. Respir. Med..

[18] Patel M (2014). Predictors of severe exacerbations, poor asthma control, and beta-agonist overuse for patients with asthma. J. Allergy Clin. Immunol. Pract..

[19] Tattersfield AE (1999). Exacerbations of asthma: a descriptive study of 425 severe exacerbations. The FACET International Study Group. Am. J. Respir. Crit. Care Med..

[20] Lee JH (2006). Gender differences in IgE-mediated allergic asthma in the epidemiology and natural history of asthma: Outcomes and Treatment Regimens (TENOR) study. J. Asthma.

[21] Adachi, M. et al. Asthma control and quality of life in a real-life setting: a cross-sectional study of adult asthma patients in Japan (ACQUIRE-2). *J. Asthma* **56**, 1016–1025 (2019). 10.1080/02770903.2018.1514628 30252543

[22] Guilbert TW (2011). Asthma that is not well-controlled is associated with increased healthcare utilization and decreased quality of life. J. Asthma.

[23] Yabe D (2015). Use of the Japanese health insurance claims database to assess the risk of acute pancreatitis in patients with diabetes: comparison of DPP-4 inhibitors with other oral antidiabetic drugs. Diabetes Obes. Metab..

[24] Furukawa TA (2013). Prescription patterns following first-line new generation antidepressants for depression in Japan: a naturalistic cohort study based on a large claims database. J. Affect Disord..

[25] Japan Medical Data Center (JMDC). https://www.jmdc.co.jp/en/about/database.html (2019).

[26] Raebel MA, Schmittdiel J, Karter AJ, Konieczny JL, Steiner JF (2013). Standardizing terminology and definitions of medication adherence and persistence in research employing electronic databases. Med. Care.

[27] Stensrud MJ, Hernán MA (2020). Why test for proportional hazards?. JAMA.

[28] Japan Medical Data Center (JMDC). Features of JMDC Claims Database. https://www.jmdc.co.jp/pharma/database.html (2018).

[29] Kimura S, Sato T, Ikeda S, Noda M, Nakayama T (2010). Development of a database of health insurance claims: standardization of disease classifications and anonymous record linkage. J. Epidemiol..

[30] Jones A (2002). Prospective, placebo-controlled trial of 5 vs 10 days of oral prednisolone in acute adult asthma. Respir. Med..

[31] Price D (2013). Real-life comparison of beclometasone dipropionate as an extrafine- or larger-particle formulation for asthma. Respir. Med..

[32] van Aalderen WM (2015). Small-particle inhaled corticosteroid as first-line or step-up controller therapy in childhood asthma. J. Allergy Clin. Immunol. Pract..

