# Supplementary material for: Uncontrolled asthma: a retrospective cohort study in Japanese patients newly prescribed with medium-/high-dose ICS/LABA
Source: NPJ Prim Care Respir Med. 2021 Mar 2;31:12. doi: 10.1038/s41533-021-00222-2 (PMC7925674; doi:10.1038/s41533-021-00222-2)
Supplement: Supplementary file 1 — Supplementary Information [file 41533_2021_222_MOESM1_ESM.pdf]

Supplementary Information file

Uncontrolled asthma: a retrospective cohort study in Japanese patients newly prescribed with medium/high-dose ICS/LABA

Hiromasa Inoue[1], Ki Lee Milligan[2], Aine McConnon[3], Hajime Yoshisue[4], Emil Loefroth[5], Martin McSharry[6], Akihito Yokoyama[7], Masakazu Ichinose[8]

[1]Department of Pulmonary Medicine, Kagoshima University, Kagoshima, Japan; [2]Novartis Pharma AG, Basel, Switzerland; [3]Novartis Ireland Limited, Dublin, Ireland; [4]Novartis Pharma K.K. Tokyo, Japan; [5]Novartis Sverige AB, Kista, Sweden; [6]OptumRx, Dublin, Ireland [7]Department of Respiratory Medicine and Allergology, Kochi Medical School, Kochi University, Kochi, Japan; [8]Osaki Citizen Hospital, Osaki, Japan

Supplementary Figure 1. Patient disposition according to GINA [1]

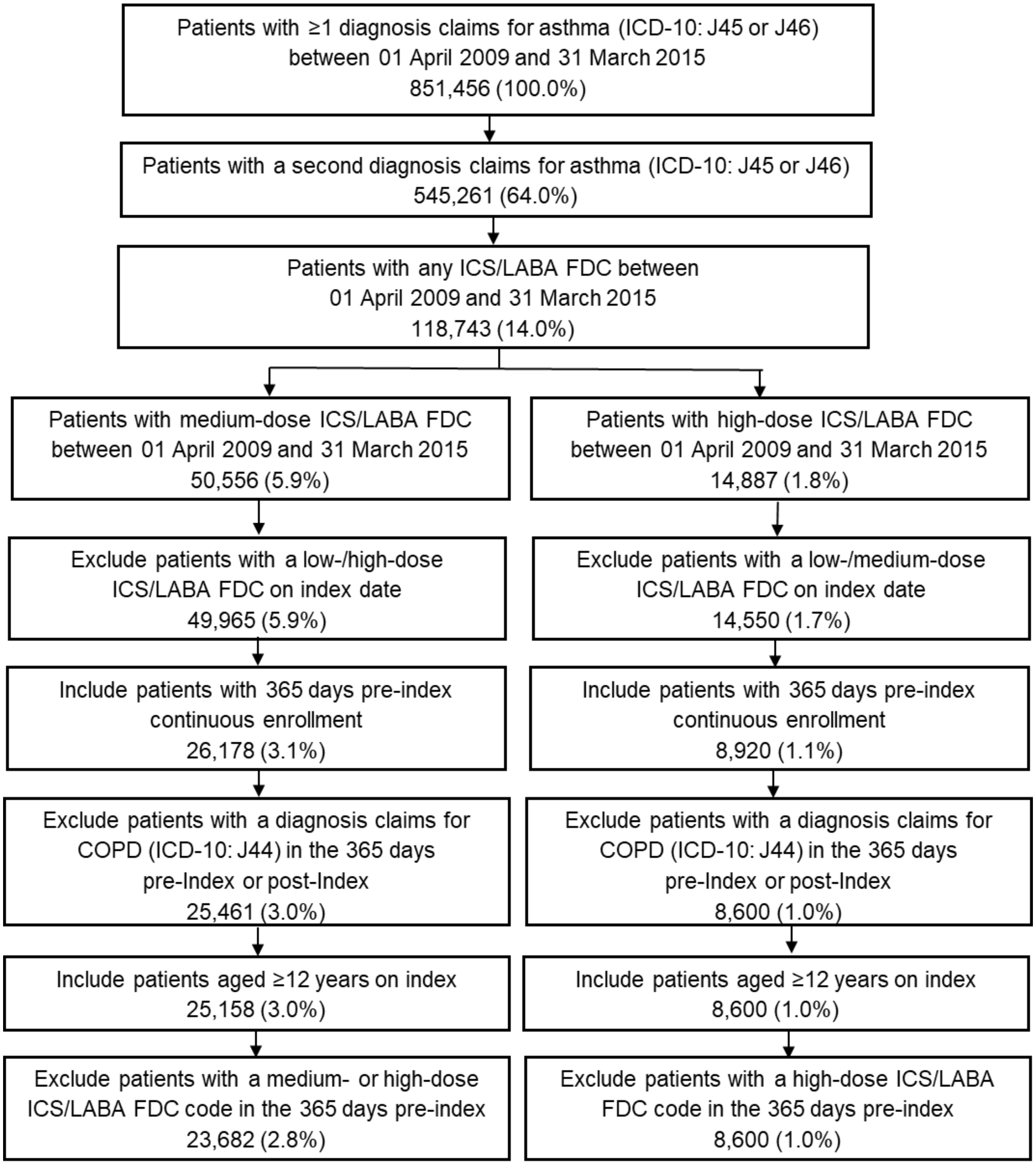

FDC, fixed-dose combination; GINA, Global Initiative for Asthma; ICS, inhaled corticosteroid; LABA, long-acting β_2-agonist

Supplementary Table 1. Baseline demographics and clinical characteristics (cohorts defined according to JGL[2])

Characteristics	Medium-dose ICS/LABA cohort (N = 24,937)	High-dose ICS/LABA cohort (N = 8,661)
Sex		
Male	11,498 (46.1%)	4,182 (48.3%)
Female	13,439 (53.9%)	4,479 (51.7%)
Age at index date*, years		
Mean ± SD	38.9 ± 13.31	40.8 ± 11.94
Range	12–75	12–75
Median (IQR)	39.0 (31.0–48.0)	40.0 (33.0–49.0)
Index year		
2010	2,341 (9.4%)	531 (6.1%)
2011	4,225 (16.9%)	1,018 (11.8%)
2012	5,354 (21.5%)	1,306 (15.1%)
2013	5,064 (20.3%)	1,562 (18.0%)
2014	6,732 (27.0%)	3,308 (38.2%)
2015	1,221 (4.9%)	936 (10.8%)
Smoking status		
Habitual smoker	2,260 (9.1%)	840 (9.7%)
Non-habitual smoker	8,072 (32.4%)	3,131 (36.2%)
Missing/Unknown	14,605 (58.6%)	4,690 (54.2%)
BMI, kg/m^2		
Mean ± SD	23.3 ± 4.02	23.5 ± 4.14
Range	13.5–54.9	14.0–52.0
Median (IQR)	22.7 (20.5–25.3)	22.8 (20.6–25.6)
Asthma drugs in the 1-year pre-index		
Single low-dose ICS	800 (3.2%)	289 (3.3%)
Single medium-dose ICS	280 (1.1%)	139 (1.6%)
Single high-dose ICS	31 (0.1%)	35 (0.4%)
LABA	4,153 (16.7%)	1,373 (15.9%)
Low ICS/LABA free dose combination	286 (1.2%)	118 (1.4%)
Medium ICS/LABA free dose combination	95 (0.4%)	49 (0.6%)
High ICS/LABA free dose combination	13 (0.1%)	21 (0.2%)
Low-dose ICS/LABA FDC	2,575 (10.3%)	1,195 (13.8%)
Medium-dose ICS/LABA FDC	0	2,277 (26.3%)
High-dose ICS/LABA FDC	0	0
LAMA	51 (0.2%)	31 (0.4%)
LTRA	7,878 (31.6%)	3,412 (39.4%)
SABA	4,266 (17.1%)	2,100 (24.5%)
SAMA	4 (0.0%[#])	7 (0.1%)
Theophylline	3,919 (15.7%)	1,864 (21.5%)
Maintenance systemic corticosteroids	117 (0.5%)	54 (0.6%)

Number of asthma exacerbations in the 1-year pre-index		
Mean ± SD	0.2 ± 0.75	0.3 ± 1.00
Range	0–23	0–25
Median (IQR)	0.0 (0.0–0.0)	0.0 (0.0–0.0)
Exacerbation history in the 1-year pre-index		
0	22,240 (89.2%)	7,302 (84.3%)
≥1	2,697 (10.8%)	1,359 (15.7%)
Concomitant medications in the 1-year pre-index		
β-blockers	403 (1.6%)	139 (1.6%)
NSAIDs	5,204 (20.9%)	2,059 (23.8%)
Acetaminophen	8,008 (32.1%)	2,608 (30.1%)
Comorbidities		
Atrial fibrillation or other cardiac arrhythmias	1,684 (6.8%)	690 (8.0%)
Anaphylaxis	98 (0.4%)	45 (0.5%)
Anxiety/depression	1,438 (5.8%)	571 (6.6%)
Diabetes	901 (3.6%)	396 (4.6%)
Eczema	10,874 (43.6%)	3,966 (45.8%)
GERD	3,686 (14.8%)	1,717 (19.8%)
Heart failure	1,173 (4.7%)	517 (6.0%)
Ischemic heart disease	238 (1.0%)	89 (1.0%)
Psoriasis	213 (0.9%)	84 (1.0%)
Rhinitis/rhinosinusitis	18,991 (76.2%)	6,798 (78.5%)
Rhinitis/rhinosinusitis – Allergic	17,266 (69.2%)	6,234 (72.0%)
Rhinitis/rhinosinusitis - Non-allergic	20 (0.1%)	14 (0.2%)
Rhinitis/rhinosinusitis - Inconclusive	966 (3.9%)	378 (4.4%)
Sleep apnea	457 (1.8%)	220 (2.5%)
Charlson Comorbidities		
Myocardial infarction	578 (2.3%)	258 (3.0%)
Congestive heart failure	1,212 (4.9%)	531 (6.1%)
Peripheral vascular disease	1,113 (4.5%)	433 (5.0%)
Cerebrovascular disease	2,013 (8.1%)	808 (9.3%)
Dementia	17 (0.1%)	6 (0.1%)
Chronic pulmonary disease	24,910 (99.9%)	8,659 (100.0%)
Rheumatic disease	1,290 (5.2%)	532 (6.1%)
Peptic ulcer disease	4,240 (17.0%)	1,741 (20.1%)
Mild liver disease	5,803 (23.3%)	2,214 (25.6%)
Diabetes without chronic complication	206 (0.8%)	123 (1.4%)
Diabetes with chronic complication	500 (2.0%)	217 (2.5%)
Hemiplegia or paraplegia	57 (0.2%)	27 (0.3%)
Renal disease	206 (0.8%)	87 (1.0%)
Any malignancy, including lymphoma and leukemia, except malignant neoplasm of skin	5,284 (21.2%)	2,048 (23.7%)
Moderate or severe liver disease	38 (0.2%)	14 (0.2%)
Metastatic solid tumor	335 (1.3%)	163 (1.9%)

AIDS/HIV	0	0
Charlson Comorbidity Index		
Mean ± SD	2.2 ± 1.85	2.4 ± 2.03
Range	0–17	0–16
Median (IQR)	1.0 (1.0–3.0)	2.0 (1.0–3.0)

Data presented as n (%) unless otherwise specified.
*Index date is defined as date of first medium- or high-dose ICS/LABA prescription in the identification period; #0.02%
AIDS, acquired immunodeficiency syndrome; BMI, body mass index; FDC, fixed-dose combination; GERD, gastroesophageal reflux disease; HIV, human immunodeficiency virus; ICS, inhaled corticosteroid; IQR, interquartile range; LABA, long-acting β_2-agonist; LAMA, long-acting muscarinic antagonist; LTRA, leukotriene receptor antagonist; NSAIDs, non-steroidal anti-inflammatory drugs; SABA, short-acting β_2-agonist; SAMA, short-acting muscarinic antagonist

Supplementary Table 2. Baseline demographics and clinical characteristics (cohorts defined according to GINA[1])

Characteristics	Medium-dose ICS/LABA cohort (N = 23,682)	High-dose ICS/LABA cohort (N = 8,600)
Sex		
Male	10,820 (45.7%)	4,144 (48.2%)
Female	12,862 (54.3%)	4,456 (51.8%)
Age at index date, years		
Mean ± SD	40.3 ± 12.19	41.0 ± 11.74
Range	12–75	16–75
Median (IQR)	40.0 (32.0–48.0)	40.0 (33.0–49.0)
Index year		
2010	2,253 (9.5%)	528 (6.1%)
2011	4,027 (17.0%)	1,013 (11.8%)
2012	5,079 (21.5%)	1,293 (15.0%)
2013	4,811 (20.3%)	1,551 (18.0%)
2014	6,379 (26.9%)	3,286 (38.2%)
2015	1,133 (4.8%)	929 (10.8%)
Smoking status		
Habitual smoker	2,260 (9.5%)	840 (9.8%)
Non-habitual smoker	8,072 (34.1%)	3,131 (36.4%)
Missing/Unknown	13,350 (56.4%)	4,629 (53.8%)
BMI, kg/m^2		
Mean ± SD	23.3 ± 4.02	23.5 ± 4.14
Range	13.5–54.9	14.0–52.0
Median (IQR)	22.7 (20.5–25.3)	22.8 (20.6–25.6)
Asthma drugs in the 1-year pre-index		
Single low-dose ICS	771 (3.3%)	287 (3.3%)
Single medium-dose ICS	268 (1.1%)	135 (1.6%)
Single high-dose ICS	31 (0.1%)	34 (0.4%)
LABA	3,781 (16.0%)	1,360 (15.8%)
Low ICS/LABA free dose combination	263 (1.1%)	118 (1.4%)
Medium ICS/LABA free dose combination	90 (0.4%)	49 (0.6%)
High ICS/LABA free dose combination	13 (0.1%)	21 (0.2%)
Low-dose ICS/LABA FDC	2,546 (10.8%)	1,195 (13.9%)
Medium-dose ICS/LABA FDC	37 (0.2%)	2,252 (26.2%)
High-dose ICS/LABA FDC	0	0
LAMA	51 (0.2%)	31 (0.4%)
LTRA	7,213 (30.5%)	3,373 (39.2%)
SABA	3,958 (16.7%)	2,078 (24.2%)
SAMA	4 (0.0%[#])	7 (0.1%)
Theophylline	3,761 (15.9%)	1,855 (21.6%)
Maintenance systemic corticosteroids	117 (0.5%)	54 (0.6%)

Number of asthma exacerbations in the 1-year pre-index		
Mean ± SD	0.2 ± 0.74	0.3 ± 1.00
Range	0–23	0–25
Median (IQR)	0.0 (0.0–0.0)	0.0 (0.0–0.0)
Exacerbation history in the 1-year pre-index		
0	21,146 (89.3%)	7,248 (84.3%)
≥1	2,536 (10.7%)	1,352 (15.7%)
Concomitant medications in the 1-year pre-index		
β-blockers	403 (1.7%)	139 (1.6%)
NSAIDs	4,742 (20.0%)	2,032 (23.6%)
Acetaminophen	7,210 (30.5%)	2,570 (29.9%)
Comorbidities		
Atrial fibrillation or other cardiac arrhythmias	1,655 (7.0%)	686 (8.0%)
Anaphylaxis	91 (0.4%)	45 (0.5%)
Anxiety/depression	1,427 (6.0%)	569 (6.6%)
Diabetes	895 (3.8%)	396 (4.6%)
Eczema	10,119 (42.7%)	3,928 (45.7%)
GERD	3,675 (15.5%)	1,719 (20.0%)
Heart failure	1,165 (4.9%)	515 (6.0%)
Ischemic heart disease	236 (1.0%)	89 (1.0%)
Psoriasis	209 (0.9%)	84 (1.0%)
Rhinitis/rhinosinusitis	17,845 (75.4%)	6,741 (78.4%)
Rhinitis/rhinosinusitis – Allergic	16,177 (68.3%)	6,184 (71.9%)
Rhinitis/rhinosinusitis - Non-allergic	19 (0.1%)	14 (0.2%)
Rhinitis/rhinosinusitis - Inconclusive	885 (3.7%)	377 (4.4%)
Sleep apnea	455 (1.9%)	219 (2.6%)
Charlson Comorbidities		
Myocardial infarction	571 (2.4%)	258 (3.0%)
Congestive heart failure	1,201 (5.1%)	529 (6.2%)
Peripheral vascular disease	1,098 (4.6%)	433 (5.0%)
Cerebrovascular disease	1,997 (8.4%)	808 (9.4%)
Dementia	17 (0.1%)	6 (0.1%)
Chronic pulmonary disease	23,659 (99.9%)	8,598 (100.0%)
Rheumatic disease	1,272 (5.4%)	533 (6.2%)
Peptic ulcer disease	4,207 (17.8%)	1,741 (20.2%)
Mild liver disease	5,673 (24.0%)	2,207 (25.7%)
Diabetes without chronic complication	204 (0.9%)	123 (1.4%)
Diabetes with chronic complication	500 (2.1%)	218 (2.5%)
Hemiplegia or paraplegia	55 (0.2%)	27 (0.3%)
Renal disease	202 (0.9%)	87 (1.0%)
Any malignancy, including lymphoma and leukemia, except malignant neoplasm of skin	5,269 (22.3%)	2,048 (23.8%)
Moderate or severe liver disease	36 (0.2%)	13 (0.2%)
Metastatic solid tumor	334 (1.4%)	163 (1.9%)

AIDS/HIV	0	0
Charlson Comorbidity Index		
Mean ± SD	2.3 ± 1.88	2.4 ± 2.03
Range	0–17	0–16
Median (IQR)	2.0 (1.0–3.0)	2.0 (1.0–3.0)

Data presented as n (%) unless otherwise specified

*Index date is defined as date of first medium- or high-dose ICS/LABA prescription in the identification period; #0.02%

BMI, body mass index; FDC, fixed-dose combination; GERD, gastroesophageal reflux disease; GINA, Global Initiative for Asthma; ICS, inhaled corticosteroid; IQR, interquartile range; LABA, long-acting β_2-agonist; LAMA, long-acting muscarinic antagonist; LTRA, leukotriene receptor antagonist; NSAIDs, non-steroidal anti-inflammatory drugs; SABA, short-acting β_2-agonist; SAMA, short-acting muscarinic antagonist

Supplementary Table 3. Healthcare resource utilization in the post-index period in medium-dose and high-dose ICS/LABA cohorts

HCRU event	Medium-dose ICS/LABA cohort			High-dose ICS/LABA cohort		
	Controlled asthma	Uncontrolled asthma	*P*-value*	Controlled asthma	Uncontrolled asthma	*P*-value*
Asthma specific						
Outpatient visits	326.0 (322.03, 330.01)	580.4 (567.44, 593.76)	<0.0001	355.5 (347.91, 363.31)	686.44 (658.71, 715.35)	<0.0001
Hospitalizations	0	0	1.0000	0	0	1.0000
Outpatient ER	55.7 (54.04, 57.34)	91.7 (86.59, 97.05)	<0.0001	60.1 (56.99, 63.32)	148.4 (135.76, 162.12)	<.0001
Length of stay of hospitalization	0	0	1.0000	0	0	1.0000
Prescriptions	872.1 (865.55, 878.62)	1,545.7 (1,542.42, 1,567.36)	<0.0001	1,207.9 (1,193.75, 1,222.13)	2,323.5 (2,272.00, 2,376.19)	<0.0001
All-cause (excl. asthma specific)						
Outpatient visits	522.4 (517.33, 527.44)	699.0 (684.69, 713.56)	<0.0001	529.1 (519.75, 538.53)	771.0 (741.53, 801.55)	<0.0001
Hospitalizations	3.2 (2.83, 3.62)	5.2 (4.09, 6.61)	0.0004	2.5 (1.91, 3.21)	6.7 (4.40, 10.16)	<0.0001
Outpatient ER	66.7 (64.94, 68.56)	86.2 (81.24, 91.38)	<0.0001	74.4 (70.91, 77.96)	104.9 (94.38, 116.55)	<0.0001
Length of stay of hospitalization	28.4 (27.28, 29.64)	32.8 (29.85, 36.12)	0.0066	21.8 (19.96, 23.78)	55.6 (48.13, 64.31)	<0.0001
Prescriptions	2,228.9 (2218.50, 2,239.39)	2,584.1 (2,556.47, 2,611.99)	<0.0001	2,363.1 (2,343.34, 2,383.04)	2,788.3 (2,731.85, 2,845.98)	<0.0001

Data presented as rate per 100 person-years (95% CI); *Wald test from Poisson model testing parameter for asthma control
P-Values: Wald test from Poisson testing parameter for asthma control
ER, emergency room; HCRU, healthcare resource utilization; ICS, inhaled corticosteroid; LABA, long-acting β_2-agonist

Supplementary Table 4. Annualized healthcare costs in patients achieving asthma control versus uncontrolled asthma (cohorts defined according to JGL[2])

Characteristic	Medium-dose ICS/LABA cohort (N = 24,937)			High-dose ICS/LABA cohort (N = 8,661)		
	Controlled asthma (n = 19,230)	Uncontrolled asthma (n = 5,707)	*P*-value*	Controlled asthma (n = 6,868)	Uncontrolled asthma (n = 1,793)	*P*-value*
All-cause (excl. asthma specific) healthcare costs, Yen						
Hospitalizations						
Mean ± SD	14,130.3 ± 273,310.01	28,194.8 ± 570,105.87		10,188.2 ± 225,997.38	45,987.0 ± 814,231.22	
Range	0–16,054,143	0–31,750,307	0.7417	0–11,656,518	0–28,221,136	0.1997
Median (IQR)	0.0 (0.0–0.0)	0.0 (0.0–0.0)		0.0 (0.0–0.0)	0.0 (0.0–0.0)	
Outpatient ER						
Mean ± SD	7,288.0 ± 56,169.69	9,652.4 ± 70,612.07		7,804.8 ± 46,751.77	9,046.1 ± 68,796.45	0.0075
Range	0–4,613,079	0–2,098,750	<0.0001	0–1,434,284	0–1,641,081	
Median (IQR)	0.0 (0.0–0.0)	0.0 (0.0–0.0)		0.0 (0.0–0.0)	0.0 (0.0–0.0)	
Outpatient visits						
Mean ± SD	50,494.8 ± 170,057.20	69,386.0 ± 255,951.84		49,776.3 ± 156,970.75	70,302.3 ± 244,605.87	
Range	0–5,822,450	0–6,257,061	0.0002	0–4,882,118	0–4,575,994	0.0038
Median (IQR)	4,593.4 (0.0–46,520.0)	0.0 (0.0–51,337.4)		0.0 (0.0–47,009.2)	0.0 (0.0–49,658.4)	
Medications prescription						
Mean ± SD	27,200.8 ± 121,544.87	29,711.2 ± 166,018.61		28,938.7 ± 120,027.43	25,707.3 ± 115,613.08	<0.0001
Range	0–8,069,623	0–7,866,923	<0.0001	0–4,043,387	0–2,159,179	
Median (IQR)	1,119.8 (0.0–17,058.9)	0.0 (0.0–12,519.5)		0.0 (0.0–17,116.0)	0.0 (0.0–7,675.8)	
Total costs (excl. asthma specific)						
Mean ± SD	99,113.9 ± 394,522.37	136,944.4 ± 684,472.59	<0.0001	96,708.0 ± 357,972.06	151,042.6 ± 882,978.56	<0.0001

Range	0–17,135,902	0–31,750,307		0–13,282,889	0–28,497,541	
Median (IQR)	15,537.1 (0.0–84,475.8)	6,145.0 (0.0–89,671.9)		10,893.5 (0.0–84,166.6)	0.0 (0.0–92,569.6)	
**Asthma-specific healthcare costs, Yen**						
Hospitalizations						
Mean ± SD	0 ± 0	0 ± 0		0 ± 0	0 ± 0	
Range	0–0	0–0	1.0000	0–0	0–0	1.000
Median (IQR)	0.0 (0.0–0.0)	0.0 (0.0–0.0)		0.0 (0.0–0.0)	0.0 (0.0–0.0)	
Outpatient ER						
Mean ± SD	10,701.6 ± 71,752.48	23,068.0 ± 292,682.04		11,462.8 ± 64,897.85	25,044.7 ± 143,601.53	
Range	0–4,932,870	0–17,731,700	0.6945	0–2,139,813	0–2,950,417	0.0826
Median (IQR)	0.0 (0.0–0.0)	0.0 (0.0–0.0)		0.0 (0.0–0.0)	0.0 (0.0–0.0)	
Outpatient visits						
Mean ± SD	58,146.6 ± 171,483.15	121,354.5 ± 498,237.41		68,968.3 ± 214,460.19	146,679.9 ± 531,025.77	
Range	0–8,230,142	0–17,731,700	<0.0001	0–8,661,551	0–8,643,200	0.0680
Median (IQR)	9,030.7 (0.0–59,426.6)	16,022.1 (0.0–89,268.6)		9,205.9 (0.0–65,528.9)	8,572.8 (0.0–99,071.4)	
Medications prescription						
Mean ± SD	90,215.8 ± 124,535.91	161,620.5 ± 358,971.59		125,022.8 ± 171,300.74	218,072.9 ± 507,082.21	
Range	2,919–3,999,013	0–10,628,070	<0.0001	3,144–3,637,773	0–12,596,223	0.4586
Median (IQR)	85,596.0 (12,630.4–132,802.6)	75,546.8 (16,448.0–176,899.9)		99,678.9 (21,409.8–167,366.3)	90,032.5 (14,928.2–228,755.9)	
Total costs (asthma specific)						
Mean ± SD	159,064.0 ± 258,518.86	306,043.0 ± 849,291.91		205,453.8 ± 313,437.41	389,797.6 ± 808,154.43	
Range	2,919–9,963,475	0–38,066,836	<0.0001	3,144–8,898,520	0–12,596,223	<0.0001
Median (IQR)	96,645.0 (27,072.6–203,093.7)	145,410.8 (36,734.8–307,768.0)		128,329.9 (41,451.5–253,407.8)	176,372.6 (28,799.1–425,371.0)	
**Total costs, Yen**						
Mean ± SD	258,177.9 ± 490,068.51	442,987.3 ± 1,119,942.39	<0.0001	302,161.8 ± 497,373.74	540,840.2 ± 1,221,917.13	<0.0001

Range	3,373–17,586,184	0–38,066,836		3,144–13,669,940	0–28,772,967
Median (IQR)	150,921.2 (83,791.0–292,405.1)	208,827.6 (88,061.7–422,795.1)		181,646.1 (99,226.5–347,110.4)	241,928.1 (80,482.1–548,702.8)

Data presented as mean ± SD. *Wilcoxon rank sum test
Annualized costs = costs during follow-up period/(duration of follow-up period/365)
ER, emergency room; JGL, Japanese asthma guidelines; IQR, interquartile range

Supplementary Table 5. Asthma control in medium-dose and high-dose ICS/LABA cohort (cohorts defined according to GINA [1])

	Medium-dose ICS/LABA cohort (N = 23,682)	High-dose ICS/LABA cohort (N = 8,600)
Patients with uncontrolled asthma	5,437 (23.0%)	1,782 (20.7%)
Reasons for uncontrolled asthma		
Episode of exacerbation		
Moderate exacerbation	1,401 (5.9%)	672 (7.8%)
Severe exacerbation	194 (0.8%)	92 (1.1%)
Treatment step-up		
ICS increased dose	490 (2.1%)	0
Addition of LAMA	10 (0.0%*)	11 (0.1%)
Addition of LTRA	1,777 (7.5%)	491 (5.7%)
Addition of theophylline	571 (2.4%)	211 (2.5%)
Maintenance systemic corticosteroids	75 (0.3%)	31 (0.4%)
Addition of more than one asthma controller, including LTRA	190 (0.8%)	54 (0.6%)
Addition of more than one asthma controller, excluding LTRA	23 (0.1%)	0
High-dose SABA	519 (2.2%)	186 (2.2%)
Use of adrenaline	573 (2.4%)	168 (2.0%)
Time to first episode of uncontrolled asthma control, median, days	31.0	21.0
Time to first episode of exacerbation, median, days	11.0	8.0

Data presented as n (%), unless otherwise specified. *0.04%
ICS, inhaled corticosteroid; LABA, long-acting β_2-agonist; LAMA, long-acting muscarinic antagonist; LTRA, leukotriene receptor antagonist; SABA, short-acting β_2-agonist

Supplementary Table 6. Demographic and clinical characteristics of patients achieving asthma control versus patients with uncontrolled asthma in the post-index period (cohorts defined according to GINA[1])

Characteristic	Medium-dose ICS/LABA cohort (N = 23,682)			High-dose ICS/LABA cohort (N = 8,600)		
	Controlled asthma (n = 18,245)	Uncontrolled asthma (n = 5,437)	*P*-value	Controlled asthma (n = 6,818)	Uncontrolled asthma (n = 1,782)	*P*-value
Sex						
Male	8,481 (46.5%)	2,339 (43.0%)	<0.0001*	3,359 (49.3%)	785 (44.1%)	<0.0001*
Female	9,764 (53.5%)	3,098 (57.0%)		3,459 (50.73%)	997 (56.0%)	
Age at index date, years						
Mean ± SD	40.2 ± 12.12	40.7 ± 12.41	0.0074#	40.9 ± 11.74	41.6 ± 11.74	0.0348#
Range	16–75	12–75		16–75	16–74	
Median (IQR)	40.0 (32.0–48.0)	40.0 (32.0–49.0)		40.0 (33.0–48.0)	41.0 (33.0–49.0)	
BMI, kg/m^2						
Mean ± SD	23.3 ± 4.00	23.2 ± 4.08	0.3220#	23.5 ± 4.07	23.6 ± 4.43	0.7950#
Range	13.5–54.9	14.6–46.6		15.3–52.0	14.0–42.4	
Median (IQR)	22.7 (20.5–25.3)	22.6 (20.4–25.3)		22.9 (20.7–25.6)	22.7 (20.4–25.9)	
Index year						
2010	1,659 (9.1%)	594 (10.9%)	<0.0001*	372 (5.5%)	156 (8.8%)	<0.0001*
2011	2,987 (16.4%)	1,040 (19.1%)		699 (10.3%)	314 (17.6%)	
2012	3,941 (21.6%)	1,138 (20.9%)		1,010 (14.8%)	283 (15.9%)	
2013	3,720 (20.4%)	1,091 (20.1%)		1,238 (18.2%)	313 (17.6%)	
2014	4,950 (27.1%)	1,429 (26.3%)		2,649 (38.9%)	637 (35.8%)	
2015	988 (5.4%)	145 (2.7%)		850 (12.5%)	79 (4.4%)	
Asthma exacerbations in 1-year pre-index						
Mean ± SD	0.1 ± 0.56	0.3 ± 1.16	<0.0001#	0.2 ± 0.69	0.6 ± 1.70	<0.0001#
Range	0–12	0–23		0–16	0–25	
Median (IQR)	0.0 (0.0–0.0)	0.0 (0.0–0.0)		0.0 (0.0–0.0)	0.0 (0.0–1.0)	
Patients with asthma exacerbations in 1-year pre-index						
0	16,582 (90.9%)	4,564 (83.9%)	<0.0001#	5,921 (86.8%)	1,327 (74.5%)	<0.0001#
≥1	1,770 (9.2%)	927 (16.2%)		897 (13.2%)	455 (25.5%)	

Asthma drugs in 1-year pre-index						
LAMA	35 (0.2%)	16 (0.3%)	0.1526*	21 (0.3%)	10 (0.6%)	0.1123*
LTRA	5,437 (29.8%)	1,776 (32.7%)	<0.0001*	2,619 (38.41%)	754 (42.31%)	0.0027*
SABA	2,910 (16.0%)	1,048 (19.3%)	<0.0001*	1,535 (22.5%)	543 (30.5%)	<0.0001*
Theophylline	2,803 (15.4%)	958 (17.6%)	<0.0001*	1,391 (20.4%)	464 (26.0%)	<0.0001*
Maintenance systemic corticosteroids	34 (0.2%)	83 (1.5%)	<0.0001*	22 (0.3%)	32 (1.8%)	<0.0001*
Concomitant medications in 1-year pre-index						
NSAIDs	3,480 (19.1%)	1,262 (23.2%)	<0.0001*	1,561 (22.9%)	471 (26.4%)	0.0018*
Beta-blockers	300 (1.6%)	103 (1.9%)	0.2107*	102 (1.5%)	37 (2.1%)	0.0837*
Acetaminophen	5,418 (29.7%)	1,792 (33.0%)	<0.0001*	1,990 (29.2%)	580 (32.6%)	0.0058*
Comorbidities						
Atrial fibrillation or other cardiac arrhythmias	1,192 (6.5%)	463 (8.5%)	<0.0001*	536 (7.9%)	150 (8.4%)	0.4405*
Anaphylaxis	56 (0.3%)	35 (0.6%)	0.0004*	31 (0.5%)	14 (0.8%)	0.0847*
Eczema	7,644 (41.9%)	2,475 (45.5%)	<0.0001*	3,063 (44.9%)	865 (48.5%)	0.0064*
GERD	2,676 (14.7%)	999 (18.4%)	<0.0001*	1,325 (19.4%)	394 (22.1%)	0.0119*
Heart failure	829 (4.5%)	336 (6.2%)	<0.0001*	402 (5.9%)	113 (6.3%)	0.4808*
Rhinitis/ rhinosinusitis	13,538 (74.2%)	4,307 (79.2%)	<0.0001*	5,309 (77.9%)	1,432 (80.4%)	0.0229*
Rhinitis/ rhinosinusitis – allergic	12,215 (67.0%)	3,962 (72.9%)	<0.0001*	4,855 (71.2%)	1,329 (74.6%)	0.0048[c]
Charlson Comorbidity Index						
Mean ± SD	2.2 ± 1.82	2.5 ± 2.05	<0.0001[#]	2.4 ± 1.97	2.6 ± 2.23	0.0040[#]
Range	0–17	0–16		0–16	0–16	
Median (IQR)	1.0 (1.0–3.0)	2.0 (1.0–3.0)		2.0 (1.0–3.0)	2.0 (1.0–3.0)	

Data presented as n (%), unless otherwise specified. *Chi-square test; [#]Wilcoxon rank sum test
BMI, body mass index; FDC, fixed-dose combination; GERD, gastroesophageal reflux disease; ICS, inhaled corticosteroid; IQR, interquartile range; LABA, long-acting β_2-agonist LAMA, long-acting muscarinic antagonist; LTRA, leukotriene receptor antagonist; NSAID, nonsteroidal anti-inflammatory drug; SABA, short-acting β_2-agonist

Supplementary Table 7. Low-, medium- and high-dose ICS/LABA according to JGL [2]

Drug name	Low-dose	Medium-dose	High-dose
Fluticasone propionate/salmeterol xinafoate (DPI)	200 μg/day	500 μg/day	1000 μg/day
Fluticasone propionate/salmeterol xinafoate (pMDI)	200 μg/day	500 μg/day	1000 μg/day
Fluticasone propionate/salmeterol xinafoate*	100 μg/day	200 μg/day	200 μg/day plus additional ICS
Fluticasone furoate/vilanterol trifenatate (DPI)	100 μg/day	n.a.	200 μg/day
Fluticasone propionate/formoterol fumarate (pMDI)	200 μg/day	500 μg/day	1000 μg/day
Budesonide/formoterol fumarate (DPI)	320 μg/day	640 μg/day	1280 μg/day

*Pediatric patients (aged 6 to 15 years), available only in the Japanese version of JGL, 2017. Fluticasone propionate/salmeterol xinafoate is the only ICS/LABA drug approved for children in Japan; prescriptions for other ICS/LABA drugs for patients (aged <16 years) were not included in the analysis
DPI, dry powder inhaler; ICS, inhaled corticosteroid; LABA, long-acting β_2-agonist; pMDI, pressurized metered dose inhaler

Supplementary Table 8. Low-, medium- and high-dose ICS/LABA according to GINA [1]

Drug name	Low-dose	Medium-dose	High-dose
Adults and adolescents (12 years and older)			
Beclometasone dipropionate (CFC)*	200–500	>500–1000	>1000
Beclometasone dipropionate (HFA)	100–200	>200–400	>400
Budesonide (DPI)	200–400	>400–800	>800
Ciclesonide (HFA)	80–160	>160–320	>320
Fluticasone furoate (DPI)	100	n.a.	200
Fluticasone propionate (DPI)	100–250	>250–500	>500
Fluticasone propionate (HFA)	100–250	>250–500	>500
Mometasone furoate	110–220	>220–440	>440
Triamcinolone acetonide	400–1000	>1000–2000	>2000

*Beclometasone dipropionate (CFC) is included for comparison with older literature
CFC, chlorofluorocarbon propellant; DPI, dry powder inhaler; HFA, hydrofluoroalkane propellant; N.A. not applicable

Supplementary References

1. Global Initiative for Asthma (2016) Global Initiative for Asthma. Global Strategy for Asthma Management and Prevention. Available at www.ginasthma.org. Accessed on 6 September 2019.
2. Ichinose, M. *et al.* Japanese guidelines for adult asthma 2017. *Allergol Int* **66**, 163-189, doi:10.1016/j.alit.2016.12.005 (2017).
