# Supplementary material for: Uncontrolled asthma: a retrospective cohort study in Japanese patients newly prescribed with medium-/high-dose ICS/LABA
Source: NPJ Prim Care Respir Med. 2021 Mar 2;31:12. doi: 10.1038/s41533-021-00222-2 (PMC7925674; doi:10.1038/s41533-021-00222-2)
Supplement: Supplementary file 2 — Reporting Summary [file 41533_2021_222_MOESM2_ESM.pdf]

nature research

Corresponding author(s): NPJPCRM-01077

Last updated by author(s): Jan 18, 2021

Reporting Summary

Nature Research wishes to improve the reproducibility of the work that we publish. This form provides structure for consistency and transparency in reporting. For further information on Nature Research policies, see our Editorial Policies and the Editorial Policy Checklist.

Statistics

For all statistical analyses, confirm that the following items are present in the figure legend, table legend, main text, or Methods section.

n/a	Confirmed	
☐	☒	The exact sample size (n) for each experimental group/condition, given as a discrete number and unit of measurement
☐	☒	A statement on whether measurements were taken from distinct samples or whether the same sample was measured repeatedly
☐	☒	The statistical test(s) used AND whether they are one- or two-sided<br>*Only common tests should be described solely by name; describe more complex techniques in the Methods section.*
☐	☒	A description of all covariates tested
☐	☒	A description of any assumptions or corrections, such as tests of normality and adjustment for multiple comparisons
☐	☒	A full description of the statistical parameters including central tendency (e.g. means) or other basic estimates (e.g. regression coefficient) AND variation (e.g. standard deviation) or associated estimates of uncertainty (e.g. confidence intervals)
☒	☐	For null hypothesis testing, the test statistic (e.g. F, t, r) with confidence intervals, effect sizes, degrees of freedom and P value noted<br>*Give P values as exact values whenever suitable.*
☒	☐	For Bayesian analysis, information on the choice of priors and Markov chain Monte Carlo settings
☒	☐	For hierarchical and complex designs, identification of the appropriate level for tests and full reporting of outcomes
☒	☐	Estimates of effect sizes (e.g. Cohen's d, Pearson's r), indicating how they were calculated

Our web collection on statistics for biologists contains articles on many of the points above.

Software and code

Policy information about availability of computer code

Data collection	Not applicable
Data analysis	Statistical analyses were performed using SAS version 9.4.

For manuscripts utilizing custom algorithms or software that are central to the research but not yet described in published literature, software must be made available to editors and reviewers. We strongly encourage code deposition in a community repository (e.g. GitHub). See the Nature Research guidelines for submitting code & software for further information.

Data

Policy information about availability of data

All manuscripts must include a data availability statement. This statement should provide the following information, where applicable:

- Accession codes, unique identifiers, or web links for publicly available datasets
- A list of figures that have associated raw data
- A description of any restrictions on data availability

Novartis is committed to sharing access to patient-level data and supporting documents from eligible studies with qualified external researchers. These requests are reviewed and approved by an independent review panel on the basis of scientific merit. All data provided are anonymized to respect the privacy of patients who have participated in the trial in line with applicable laws and regulations.

Field-specific reporting

Please select the one below that is the best fit for your research. If you are not sure, read the appropriate sections before making your selection.

☒ Life sciences ☐ Behavioural & social sciences ☐ Ecological, evolutionary & environmental sciences

For a reference copy of the document with all sections, see nature.com/documents/nr-reporting-summary-flat.pdf

Life sciences study design

All studies must disclose on these points even when the disclosure is negative.

Sample size	This was a retrospective analysis of Japan Medical Data Center database conducted between April 2009 to March 2015. A total of 33,598 patients identified with asthma diagnosis formed the base population of study
Data exclusions	Patients with a diagnosis of COPD (ICD-10 J44) were excluded. Patients were excluded from the medium-dose ICS/LABA cohort if they had a prescription for medium- or high-dose ICS/LABA in the pre-index period or a prescription for low- or high-dose ICS/LABA on the index date. Patients were excluded from the high-dose ICS/LABA cohort if they had a prescription for high-dose ICS/LABA in the pre-index period or a prescription for low- or medium-dose ICS/LABA on the index date.
Replication	This was a retrospective analysis and no experimental methods were setup. Data collection and analysis were the main aspects, which were repeated for all included patients from April 2009 to March 2015
Randomization	Not applicable
Blinding	Not applicable

Reporting for specific materials, systems and methods

We require information from authors about some types of materials, experimental systems and methods used in many studies. Here, indicate whether each material, system or method listed is relevant to your study. If you are not sure if a list item applies to your research, read the appropriate section before selecting a response.

Materials & experimental systems

n/a	Involved in the study	
☒	☐	Antibodies
☒	☐	Eukaryotic cell lines
☒	☐	Palaeontology and archaeology
☒	☐	Animals and other organisms
☐	☒	Human research participants
☒	☐	Clinical data
☒	☐	Dual use research of concern

Methods

n/a	Involved in the study	
☒	☐	ChIP-seq
☒	☐	Flow cytometry
☒	☐	MRI-based neuroimaging

Human research participants

Policy information about studies involving human research participants

Population characteristics	Patients aged ≥12 years at the index date with the following: evidence of 2 or more claims of asthma diagnoses as defined by the International Classification of Diseases (ICD-10) of J45 or J46 occurring between April 2009 and March 2015, newly initiated on maintenance treatment with medium- or high-dose ICS/LABA between 01 April 2009 and 31 March 2015, and at least 1 year of enrollment (continuous data available in the database) prior to the index date were included
Recruitment	Eligible patients included employees and their family members covered by their company health insurance plan. As this was retrospective analysis of patients included in JMDC database, no formal recruitment process was followed
Ethics oversight	The study was approved by the Clinical Research Review Network of the Japan Review Board, Osaka, Japan.

Note that full information on the approval of the study protocol must also be provided in the manuscript.
